# Testing an online screening tool for epilepsy surgery evaluation

**DOI:** 10.1055/s-0044-1791659

**Published:** 2024-11-11

**Authors:** Bianca Cecchele Madeira, Suzana Veiga Schönwald, Pablo Brea Winckler, Kelin Cristine Martin, Carolina Machado Torres, Jorge Wladimir Junqueira Bizzi, Marino Muxfeldt Bianchin

**Affiliations:** 1 Universidade Federal do Rio Grande do Sul, Faculdade de Medicina, Programa de Pós-Graduação em Medicina: Ciências Médicas, Porto Alegre RS, Brazil.; 2 Hospital de Clínicas de Porto Alegre, Departamento de Neurologia, Porto Alegre RS, Brazil.; 3 Hospital de Clínicas de Porto Alegre, Centro de Tratamento de Epilepsias Refratárias, Porto Alegre RS, Brazil.; 4 Universidade Federal do Rio Grande do Sul, Faculdade de Medicina, Hospital de Clínicas de Porto Alegre, Serviço de Neurocirurgia, Porto Alegre RS, Brazil.; 5 Hospital de Clínicas de Porto Alegre, Centro de Pesquisa Experimental, Laboratório Basic Research and Advanced Investigations in Neurosciences (BRAIN), Porto Alegre RS, Brazil.

**Keywords:** Clinical Decision-Making, Health Care Costs, Electroencephalography, Tomada de Decisão Clínica, Custos de Cuidados de Saúde, Eletroencefalografia

## Abstract

**Background** Epilepsy surgery is recognized for its effectiveness in controlling seizures in a significant number of patients with drug-resistant epilepsy. Despite this, there remains a notable deficiency in referring these patients for video-electroencephalogram (EEG) monitoring and surgical evaluation. Addressing this gap, the Canadian Appropriateness of Epilepsy Surgery (CASES), an online tool for epilepsy surgery evaluation ( www.epilepsycases.com ), was developed to aid physicians in the referral process of patients with refractory epilepsy to surgical assessments.

**Objective** The present study aimed to evaluate the utility of CASES in identifying candidates for epilepsy surgery, thereby facilitating clinical decision-making for patients with drug-resistant epilepsy.

**Methods** A cross-sectional analysis was conducted using the CASES platform to assess surgical candidacy among individuals with epilepsy. Participants were selected among those receiving care at the Epilepsy Clinic of the Neurology Service, Hospital de Clínicas de Porto Alegre, Brazil, over a 3-month period. The study cohort included 211 patients. Data were systematically extracted from patient medical records or collected during clinical appointments.

**Results** Of the evaluated cohort, 59.6% were identified as potential candidates for video-EEG monitoring and subsequent surgical evaluation. Factors significantly associated with recommendations for video-EEG and surgical assessment included seizure frequency, the number of antiseizure medications (ASMs) trialed, and the occurrence of drug-related adverse effects.

**Conclusion** The CASES showed significant potential in guiding recommendations for video-EEG monitoring and facilitating referrals for epilepsy surgery. This tool may not only enhance patient treatments and outcomes but also contribute to cost savings in epilepsy management in both the short and long term.

## Resumo

**Antecedentes** A cirurgia de epilepsia é reconhecida por sua eficácia no controle de crises em um número significativo de pacientes com epilepsia resistente a medicamentos. Apesar disso, persiste uma deficiência notável na referência desses pacientes para monitoramento por vídeo-eletroencefalograma (EEG) e avaliação cirúrgica. Para avaliar essa lacuna, a Adequação Canadense da Cirurgia de Epilepsia (Canadian Appropriateness of Epilepsy Surgery CASES, na sigla em inglês) , uma ferramenta online para avaliação de cirurgia de epilepsia ( www.epilepsycases.com ), foi desenvolvida para auxiliar médicos no processo de selecionar pacientes com epilepsia refratária para avaliações cirúrgicas.

**Objetivo** Avaliar a utilidade da CASES na identificação de candidatos para cirurgia de epilepsia, facilitando assim os processos de decisão terapêutica para pacientes com epilepsia resistente a medicamentos.

**Métodos** Foi realizada uma análise transversal usando a plataforma CASES para estimar a necessidade de avaliação cirúrgica entre indivíduos com epilepsia. Os participantes foram selecionados dentre aqueles em atendimento na Clínica de Epilepsia do Serviço de Neurologia, Hospital de Clínicas de Porto Alegre, durante um período de 3 meses. O estudo incluiu uma coorte de 211 pacientes. Os dados foram sistematicamente extraídos dos prontuários médicos dos pacientes ou coletados durante consultas clínicas.

**Resultados** Da coorte avaliada, 59,6% foram identificados como potenciais candidatos para monitoramento por vídeo-EEG e subsequente avaliação cirúrgica. Fatores significativamente associados com recomendações para vídeo-EEG e avaliação cirúrgica incluíram a frequência de crises, o número de drogas anticrise (DACs) testadas e a ocorrência de efeitos adversos relacionados aos medicamentos.

**Conclusão** A CASES mostrou um potencial significativo em orientar as recomendações para monitoramento por vídeo-EEG e facilitar o encaminhamento de pacientes para avaliação cirúrgica. Ferramentas como essas podem não apenas melhorar os tratamentos e resultados dos pacientes, mas também contribuir para economias de custo no manejo da epilepsia a curto e longo prazo.

## INTRODUCTION

Epilepsy ranks as the second most prevalent neurological disorder globally, 1 with an estimated 1.5% incidence rate in Brazil. 2 3 4 While most individuals with epilepsy achieve satisfactory seizure control through medical intervention, a substantial number of patients remains refractory to pharmacological treatments. This group constitutes approximately one third of patients, with the likelihood increasing in particular epilepsy syndromes. 1 5 6 Drug-resistant epilepsy carries a heightened risk of physical and psychiatric comorbidities, cognitive deterioration, and sudden death. 1 7 8 For these patients, more specialized measures, including surgical intervention, may be imperative to manage seizures effectively. 9 10 11 12 In specific patient subsets, epilepsy surgery (ES) demonstrates exceptional efficacy, supported by level-one evidence from prospective randomized controlled trials indicating ES's superiority over pharmacotherapy in treatment-resistant mesial temporal lobe epilepsy. 1 13 14 15

Despite promising outcomes and widespread global experience, ES remains considerably underutilized. 16 17 18 19 Prompt and accurate identification of surgical candidates necessitates early and thorough clinical assessment. 20 21 Nevertheless, defining refractory seizure frequently encounters significant delays, 12 17 22 23 with factors such as ambiguity in eligibility criteria among referring clinicians contributing to these hold-ups. 24

The CASES tool, a web-based algorithm ( www.epilepsycases.com ), stands as a facilitator in the assessment of ES's appropriateness, aiding physicians in the preliminary screening of candidates for specialized ES centers. 25 Drawing from the RAND Corporation/University of California, Los Angeles (UCLA) appropriateness method, an expert panel judged the suitability of medical or surgical interventions across an extensive range of clinical scenarios. 26 The tool demonstrated both high usability and a substantial concordance rate of 84.6%, with clinical expert judgment, 27 and has been refined for better application among general practitioners and non-epileptologists. 28 It utilizes a multiple-choice question format anchored in established criteria for seizure intractability, including seizure frequency and the number of tried antiseizure medications (ASMs). The development process entailed constructing 2,646 theoretical scenarios, 25 categorized into *appropriate* , *uncertain* , or *inappropriate* for surgical evaluation based on resultant scores.

The present study evaluates the Canadian Appropriateness of Epilepsy Surgery (CASES) tool's applicability in identifying potential candidates for ES within a tertiary referral epilepsy center's outpatient clinic in Southern Brazil. It also examines the significant clinical variables influencing the ES's appropriateness score and the decision-making process in a practical setting. Our study aims to pave the way for the creation of innovative referral tools that streamline the process of identifying and directing patients with refractory epilepsy to specialized evaluation centers. By improving referral mechanisms, we seek to alleviate the overall impact of epilepsy, especially in the developing world, where the prevalence of the condition is notably higher. These tools, if integrated to Governmental Heath Care Systems, have the potential to facilitate access to advanced care, ultimately enhancing patients' outcomes and easing the epilepsy-related healthcare burden in underserved regions.

## METHODS

### Patient selection

Over a 3-month period, a total of 224 consecutive patients at the tertiary epilepsy outpatient clinic of the Hospital de Clínicas de Porto Alegre (HCPA) were enrolled in the study. To qualify for enrolment, individuals visiting the outpatient clinic had to be 12 years of age or older. The inclusion criteria established for the study were a confirmed diagnosis of focal epilepsy, prior assessments including electroencephalogram (EEG) and neuroimaging (either magnetic resonance imaging [MRI] or computed tomography [CT] scan), and a minimum follow-up period of 1 year at HCPA. Candidates with generalized epilepsy or episodes of non-epileptic seizures were not considered for inclusion. The ethical aspects of the present study were supervised and approved by the HCPA/GPPG Ethics Committee.

### Tools and questionnaires

During the study, a comprehensive review of patient records was undertaken, with any missing data being collected at the subsequent scheduled appointment. A thorough compilation of patient demographics and clinical data was acquired, including age, gender, disease course duration, and detailed seizure characterization—type, frequency, and intensity. Additionally, the study accounted for the quantity of ASMs each patient had trialed, coupled with an evaluation of any drug adverse effects as noted by attending physicians, and assimilated outcomes from EEG and neuroimaging studies.

We used the CASES tool ( www.toolsforepilepsy.com ). To gauge the suitability of epileptic seizure treatments, participants' responses to seven questions were requisitioned to derive an appropriateness score ranging from 1 to 9. These inquiries encompassed:

- classification of seizure into either with focal aware or focal impaired awareness seizures;
- assessment of disease chronicity (less than or more than one year);
- quantification of seizure prevalence (from seizure-free to frequent occurrences);
- evaluation of seizure impact (from non-disabling to disabling);
- the tally of ASMs previously tried;
- documentation of drug-related adverse effects;
- interpretation of prior EEG and neuroimaging findings, with a provision to substitute MRI data with CT results when needed.

All collated data were entered and preserved within a Microsoft Excel (Microsoft Corp., Redmond, WA, USA) database, ensuring integrity and ease of access for subsequent analysis.

### Statistical analysis

Statistical analyses were conducted using the SPSS for Windows software, version 16.0 (SPSS Inc., Chicago, IL, USA). The quantitative variables were expressed as mean and standard deviation (SD) values, whereas the categorical variables, as frequencies and percentages. The Student *t* -test was used for comparisons between groups for quantitative variables. Categorical variables were analyzed using the Chi-Squared or Fisher exact test, as appropriate, with results reported as odds ratios (ORs) accompanied by 95% confidence intervals (CIs). A two-tailed *p* -value < 0.05 was considered indicative of statistical significance.

For the purpose of the analysis, seizure frequency was classified into three categories: *None or Seldom* , merging the categories of seizure-free or in remission and fewer than one seizure per year; 1 to 12 seizures per year; and more than 12 seizures per year. The use of ASMs was also categorized into 3 groups: monotherapy (1 ASM), dual therapy (2 ASMs), and polytherapy (3 or more ASMs), which combined 2 categories of drug use from the CASES data.

## RESULTS

### General characteristics of the sample

Initially, the study collected data from 224 patients. After the exclusion of 13 patients—due to generalized primary epilepsy (9 patients) and pseudoseizure presence (4 patients)—a total of 211 individuals were eligible for inclusion in the analysis. The mean age of the participants was 41.3, with a standard deviation of 15.8 years, and ranged from 14 to 83 years. The cohort had a balanced gender distribution, comprising 103 females (48.8%) and 108 males (51.2%). The analysis also encompassed 7 patients known to have drug-resistant epilepsy who were on the waiting list for ES; their respective scores were as follows: 3 patients scored 9, 2 scored 7, and 1 each scored 8 and 6.

In terms of seizure frequency and type, 51.7% of the patients were seizure-free, while 22.7% experienced 1 to 12 seizures per year, and 25.6% had more than 12 seizures per year. The predominant seizure type was impaired awareness focal seizures, present in 94.8% of the cohort.

Regarding the use of ASMs, 52 patients (24.6%) had been treated with a single ASM, 66 patients (31.3%) with 2 ASMs, and 93 patients (44.1%) with 3 or more ASMs.

Most patients demonstrated abnormalities on EEG and MRI, with prevalence rates of 87.7% and 59.2%, respectively. Neuroimaging data revealed that 86 patients had normal findings, whereas pathological findings included malformations of cortical development (13 patients), ischemic stroke sequelae (25 patients), vascular malformations (4 patients), and hippocampal sclerosis (23 patients). Four patients exhibited neurocysticercosis. Additionally, 15 patients presented with more than one abnormal neuroimaging finding. A history of head trauma was noted in 14 patients.

### Epilepsy surgery appropriateness scores

**Figure 1:**
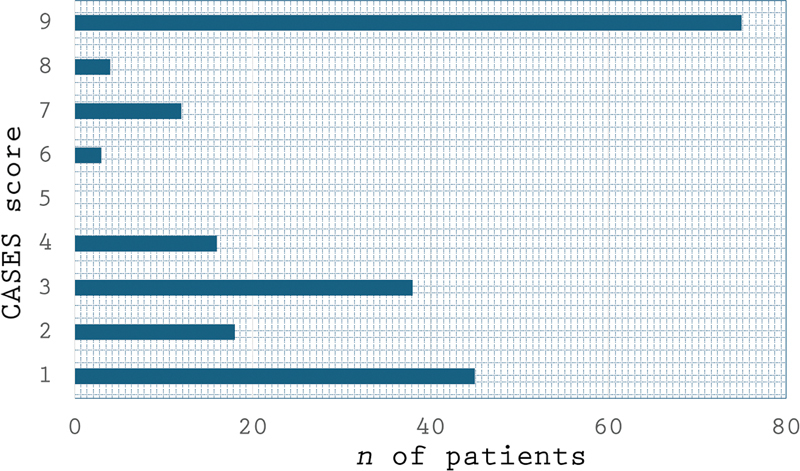
Canadian Appropriateness of Epilepsy Surgery (CASES) score distribution ( *n* = 211).

Figure 1 shows the frequency of distribution of individual ES appropriateness scores in the entire sample. The most frequently achieved score was the lowest possible (1), with 75 patients (35.5%), followed by the highest possible score (9), with 45 patients (21.3%). No patients received the intermediate score of 5. In other words, the distribution was roughly U-shaped. Almost two thirds of the patients received scores consistent with an ES evaluation referral. A hundred and one patients (47.9%) received a score consistent with very strong indication for ES evaluation, and 19 patients (9%) received a score consistent with a strong indication for ES evaluation. Ninety-one patients (34.1%) were rated as having no indication for ES evaluation referral.

### Clinical findings according to ES evaluation recommendation groups

Table 1 shows sample characteristics according to ES evaluation recommendation groups. Seizure frequency, number of ASMs and presence of drug adverse effects were significantly different among the three ES evaluation recommendation groups. Electroencephalogram findings, seizure type, and sex distribution were not significantly different among groups. There was a trend toward higher presence of abnormal neuroimage findings in Highly Indicated Surgical Evaluation (HISE) group and Indicated Surgical Evaluation (ISE) group when compared with the Not Indicated Surgical Evaluation (NISE) group.

**Table 1:** Clinical findings according to epilepsy surgery evaluation recommendation groups

Variable		HISE ( *n* = 101): n (%)	ISE ( *n* = 19): n (%)	NISE ( *n* = 91): n (%)	*p* -value
Female sex		49 (48.5%)	5 (26.3%)	49 (53.8%)	0.09
Seizure type	Focal onset aware	7 (6.9%)	1 (5.3%)	3 (3.3%)	0.5
	Focal onset impaired awareness	94 (93.1%)	18 (94.7%)	88 (96.7%)	
Seizure Frequency	None or seldom	9 (8.9%)	16 (84.2%)	84 (92.3%)	**< 0.0001**
	1–12 seizures/year	41 (40.6%)	3 (15.8%)	4 (4.4%)	
	> 12 seizures/year	51 (50.5%)	0 (0%)	3 (3.3%)	
AEDs	1	1 (1%)	1 (5.3%)	50 (54.9%)	**< 0.0001**
	2	32 (31.7%)	4 (21.1%)	30 (33%)	
	≥ 3	68 (67.3%)	14 (73.7%)	11 (12.1%)	
Drug side effects		30 (29.7%)	5 (26.3%)	5 (5.5%)	**< 0.0001**
Abnormal EEG		88 (87.1%)	17 (89.5%)	80 (87.9%)	0.9
Abnormal neuroimaging		68 (67.3%)	14 (73.7%)	43 (47.3%)	0.07

Abbreviations: AEDs, antiepileptic drugs; EEG, electroencephalogram; HISE, highly indicated surgical evaluation; ISE, indicated surgical evaluation; NISE, not indicated surgical evaluation.

Table 2 shows the chance of having an ES indication in function of the clinical variables included in the CASES tool. Variables statistically significant for the indication of surgical treatment evaluation were seizure frequency, number of drugs tested, presence of drug adverse effects, and abnormal neuroimage findings.

**Table 2:** Findings according to epilepsy surgery evaluation simplified recommendation groups

Variable		ISE ( *n* = 120): n (%)	NISE ( *n* = 91): n (%)	OR (95%CI)	*p-* value
Seizure type	Focal onset aware	8 (6.7%)	3 (3.3%)	2.09 (0.5–8.1)	0.3
	Focal onset impaired awareness	112 (93.3%)	88 (96.7%)		
Seizure frequency	Controlled seizures	25 (20.8%)	84 (92.3%)	45.6 (18.7–110.8)	**< 0.0001**
	Uncontrolled seizures	95 (79.2%)	7 (7.7%)		
AEDs	1	2 (1.7%)	50 (54.9%)	71.9 (16.7–308.9)	**< 0.0001**
	≥ 2	118 (98.3%)	41 (45.1%)		
Drug side effects	Yes	35 (29.2%)	5 (5.5%)	10.0 (3.33–20.0)	**< 0.0001**
	No	85 (70.8%)	86 (94.5%)		
Abnormal EEG		105 (87.5%)	80 (87.9%)	1.03 (0.4–2.3)	1.0
Abnormal neuroimaging		82 (68.3%)	43 (47.3%)	2.4 (1.3–4.2)	**0.003**

Abbreviations: 95%CI, 95% confidence interval; AEDs, antiepileptic drugs; EEG, electroencephalogram; ISE, indicated surgical evaluation; NISE, not indicated surgical evaluation; OR, odds ratio.

## DISCUSSION

At a tertiary referral epilepsy center associated with a university hospital in Southern Brazil, the applicability of the CASES tool—an online resource—was investigated for its utility in identifying epilepsy patients who might benefit from referral to an ES evaluation center. Additionally, the study aimed to determine which clinical variables within the CASES algorithm were most influential in decision-making processes, and their respective contributions to the appropriateness score for ES in a practical context. The key variables that guided recommendations for surgical assessment included seizure frequency, the number of ASMs trialed, and the incidence of medication adverse effects. The online tool was commended for its ease of use and consistent availability during the study.

Notably, 25 patients with well-controlled seizures were advised to seek ES evaluation, a recommendation that seemingly diverges from previous findings, 29 in which a trial of 2 ASMs was typically deemed necessary for ES consideration, unless the patient was seizure-free or lacked comprehensive investigation. This discrepancy appears because these 25 patients attained a sufficient score for evaluation through responses to other CASES questions, such as the number of ASMs tried, adverse effect experiences, and abnormal findings from ancillary tests. In contrast, seven patients who did not achieve seizure control were not recommended for surgical evaluation due to their insufficient trial of only one ASM, suggesting that an optimal medical treatment regime had not yet been fully explored. This finding aligns partially with the research by Lukmanji et al., in which 13 out of 1,044 patients were deemed ineligible for surgical assessment on the basis that they had not trialed at least 2 ASMs. 30

The neuroimaging assessment of our patient cohort is noteworthy. We adhered to the criterion that only MRI findings were to be considered valid, notwithstanding that the absence of a brain MRI did not constitute grounds for exclusion from our study. Our inclusion criteria demanded that patients have undergone some form of brain imaging, even if it was a computed tomography (CT) scan, which is more readily available than MRI in our setting. Computed tomography scans were justified on the grounds of affordability and accessibility in numerous global regions. 29 It is imperative to highlight that only findings with potential epileptogenic significance should be considered, with a requirement for individual assessment. For candidates to ES, brain MRIs ideally must be conducted with specialized protocols. 31 Non-pathological variants, or incidental findings lacking clinical significance, were not recorded as abnormalities. Neurocysticercosis has been identified both in isolation and, more commonly, in conjunction with hippocampal sclerosis. It is not uncommon for these conditions to occur simultaneously in numerous patients. 32

The CASES tool places considerable emphasis on the notion of drug-resistant epilepsy, which is mentioned twice. Initially, it arises at the outset of the assessment, where respondents must address a set of preliminary inquiries, including the confirmation of a preexisting drug-resistant epilepsy diagnosis. Affirmative responses to this, or any other initial screening question, trigger an automatic recommendation for referral to a specialized epilepsy center, irrespective of subsequent answers that contribute to the final scoring algorithm. The concept re-emerges within the variables that determine the definitive scoring for surgical candidacy, inquiring explicitly about the tally of ASMs trialed, thereby underscoring its significance in surgical decision-making. 14 33 34 Also, the disabling nature of seizures demands consideration. We postulate that seizures inherently impose a disabling effect; their mere occurrence can disrupt the patient's personal and professional life, engender stigma, inflict psychological trauma, elevate mortality risk, and other repercussions. 35 As such, categorizing all seizures as disabling appears unavoidable. Consequently, we contend that seizure severity is a subjective measure and have chosen not to include it as a variable in our statistical analysis, in reference to question number 4 of the online tool.

Patients referred for an ES evaluation are stratified into two categories based on the CASES tool: high and very high appropriateness. Each category encompasses three possible score levels, allowing for a total of six potential score levels for surgical candidates. This stratification might be useful for prioritizing patients for surgical evaluation due to the disproportion between the number of surgery candidates and the available surgical slots, a situation especially acute in developing countries. 36 Hence, the tool's utility may extend to facilitating prioritization, possibly by incorporating criteria to assess the urgency of the procedure and improve patient routing. Should the list of indications become overly extensive, a strategy to manage this could involve focusing on the most prevalent indications or honing the study's objectives to a narrower patient subset. Interestingly, the Epilepsy Surgery Grading Scale (ESGS) reflects a partial realization of this approach. 37 38 In this venue, the future integration of machine-learning algorithms may further refine assessment tools. 39

The CASES tool's clinical validation for ES evaluation has been explored. Lutz and Mayer studied 168 adult outpatients attending a regular epilepsy clinic over a 3-month period in 2012. 40 The clinical decision-making process was conducted independently of the online tool's results, with past or current referrals to a monitoring unit duly noted. According to the CASES scores, ES evaluation was deemed appropriate for 67.5% of patients, uncertain for 16.6%, and inappropriate for 16%. Out of the appropriate referrals, ES evaluations were suggested by board-certified neurologists in 43.9% of the cases. Upon excluding patients with presumed diffuse or global brain damage ( *n* = 49), the number of appropriate cases recommended for ES evaluations rose to 64.9%. The correlation between CASES ratings and clinical decisions was notably strong. The discrepancy between expert opinions and tool recommendations was attributed to various factors, including manageable seizure frequency, complex neurological malformations, psychiatric comorbidities, and patient age over 60. These factors suggest that the instrument's seizure-frequency component might only weakly reflect the overall burden of epilepsy. Furthermore, the tool may not adequately account for the epilepsy severity or for brain damage that could preclude surgery. Direct validation of the CASES tool through comparison with clinical ES indicators was not done in our study. Score distribution within the sample displayed a U-shape, indicative of two distinct outpatient subpopulations, with one subset exhibiting a milder condition less likely to reap benefits from ES evaluation. Thus, it seems that the CASES tool could assist in identifying these patients, who might be more suitably managed in a secondary-level, specialized neurology unit with lower complexity. Finally, it is important to emphasize that the CASES tool's structure inherently serves different objectives than instruments designed to predict seizure freedom post-ES. 38 However, from our perspective, merging these types of instruments could be advantageous in prioritizing candidates who have a greater likelihood of benefiting from surgical treatments. Perhaps these individuals should potentially be evaluated with precedence, especially in countries with limited resources, like Brazil.

One final comment is perhaps necessary. The limited number of ES centers in many countries face significant challenges due to the high volume of patients referred for surgical evaluation, many of whom are not suitable candidates for surgery. Ideally, these patients should not be referred for such evaluations. Unnecessary referrals cause considerable stress for patients and their families and occupy valuable evaluation slots, thereby delaying or denying access to patients who genuinely need surgical assessment. This inefficiency negatively impacts the organization and flow of ES programs, as ineligible patients restrict resources from those who could benefit from them. Given these challenges, the implementation of a web-based tool to aid public health systems in better selecting suitable candidates for ES is both timely and crucial. Thus, tools like CASES could enhance the efficiency of patient selection, ensure that only appropriate candidates undergo surgical evaluation, and ultimately improve the overall effectiveness of epilepsy surgery programs.

In conclusion, despite its established role for patients with refractory epilepsy, surgical intervention remains substantially underutilized. To bridge this gap, online screening tools might be used to streamline the assessment process for ES candidacy, thus democratizing access to specialist evaluation for healthcare providers and potentially increasing patient benefit. 41

Our research indicates that seizure frequency, the number of ASMs trialed, and the incidence of medication-related adverse effects are the primary determinants for video-EEG indications, and surgical consideration as determined by the screening tool. This underscores the tool's utility; however, there is a potential to enhance its efficiency by refining it to capture only the most pivotal data, aligning with our previous discussions. Furthermore, we think that conducting a study to validate this screening instrument for other languages, for example, Portuguese, would be invaluable, fostering more inclusive and accurate decision-making ES referral. Finally, our study's outcomes hold promise for the ongoing enhancement of the CASES algorithm. They suggest the possibility of integrating referral-prioritization mechanisms into future iterations. This evolution could significantly improve the stratification process for surgical candidates, maximizing the public health impact of these assessments, particularly in nations such as Brazil where resource allocation is critical. In other words, such a refined approach could streamline patient pathways to surgery and amplify the utility of the algorithm in effectively managing surgical caseloads in healthcare systems with limited resources.
